# No-mesh Inguinal Hernia Repair with Continuous Absorbable Sutures: Is it a Step Forward or Backward?

**DOI:** 10.4103/1319-3767.45069

**Published:** 2009-01

**Authors:** Nader Naguib, Asal ElSamerraai

**Affiliations:** Department of Surgery, Prince Charles Hospital, Wales, United Kingdom Prince Charles Hospital, Gurnos, Merthyr Tydfil-CF47 9DT- United Kingdom. E-mail: nadernaguib71@yahoo.com

Sir,

The no-mesh inguinal hernia repair, with its many different modifications, is considered in the current surgical practice as the tension repair. The tension inhibits full and effective healing of the edges. As a result, the muscle edges may pull apart causing a higher failure rate with recurrent (often larger and more complex) hernia.[1]

Desarda,[2] in the study involving 229 patients, revived the subject of nonmesh hernia repair. He used a new surgical technique using absorbable suture material for the inguinal hernia repair. The author claims a less recurrence rate and less postoperative complications with this new technique.

There are some concerns regarding this new surgical technique that need to be addressed.

There is a wide range of the duration of the follow-up for this number of patients from 6–42 months. In previous studies [Table 1] for the nonmesh inguinal hernia repair, longer periods of follow-up were used for larger numbers of patients to properly assess these procedures.

**Table 1:** Studies of no-mesh inguinal hernia repair with duration of follow-up and complication rate

Author	Type of repair	Number of patients	Follow-up period	Rate of complications	Rate of recurrence
Rutledge[3]	McVay	906	9 years	Not reported	2.0
Welsh and Alexander[4]	Shouldice	214,919	1 month to 40 years	Not reported	0.1
	Shouldice	2748	35 years	Not reported	1.5
Amid, *et al*,[5]	Lichtenstein	3250	Average of 4 years (range: 1–8 years)	Not reported	0.1
Rutkow and Robbins[6]	Rutkow	2060	NR	0.3	0.1
Nyhus[7]	Posterior iliopubic tract repair	1200	37 years		1 to 6
Felix, *et al*[8]	Transabdominal preperitoneal laparoscopic repair	733	Average of 24 months (range: 1–44 months)	13.0	0.3
	Total extraperitoneal laparoscopic repair	382	Average of 9 months (range: 1–44 months)	11.0	0.3

As regard to the surgical technique, the author states that a thin filmy layer superficial to the external oblique muscle was left undisturbed. The author fails to describe the significance of this maneuver. Furthermore, the surgical technique lacks the benefit of the tension-free repair. In fact, there is contradiction in the discussion of the procedure, as the author denies the absence of tension on the suture line (paragraph 4) while elsewhere (paragraph 6) he mentions that contraction of the muscles makes tension on the muscle strip.

The repair depends on the aponeurotic sheath of the external oblique as the only posterior layer but the original weak posterior wall has not been repaired. Suturing the edge of the upper flap to the posterior wall does not strengthen the posterior wall muscles as claimed. On the contrary, it disturbs the physiology of the abdominal wall muscles by suturing them together. So, it is not a physiological repair as claimed as the muscles run in different directions even in the inguinal canal region.

The author states that 209 patients preferred to stay back even though they were allowed to go home the same day; the author fails to give an explanation for this preference. He defined a scoring system for pain but there is no definition of the discomfort despite being experienced by most patients.

The study mentions the cost effectiveness of this outpatient technique, while there was no single patient who went home the same day. On the contrary, there is unexplained hospital stay of 8.74% patients for more than a day increasing the monetary burden for a simple procedure as hernia repair.

The author needs to be queried for the following: (1) Why should there be a long learning curve for general surgeons in other techniques and not for this one? (2) What are the risks of the dissection of inguinal canal floor that are not present in the mentioned technique?

Finally, it should be noted that it is not true that all the nonmesh repair techniques use interrupted stitches.
